# Overall negative trends for snow cover extent and duration in global mountain regions over 1982–2020

**DOI:** 10.1038/s41598-022-16743-w

**Published:** 2022-08-12

**Authors:** C. Notarnicola

**Affiliations:** EURAC Research, Bolzano, Italy

**Keywords:** Cryospheric science, Environmental impact

## Abstract

Notwithstanding the large availability of data and models, a consistent picture of the snow cover extent and duration changes in global mountain areas is lacking for long-term trends. Here, model data and satellite images are combined by using Artificial Neural Networks to generate a consistent time series from 1982 to 2020 over global mountain areas. The analysis of the harmonized time series over 38 years indicates an overall negative trend of − 3.6% ± 2.7% for yearly snow cover extent and of − 15.1 days ± 11.6 days for snow cover duration. The most affected season by negative trends is winter with an average reduction in snow cover extent of − 11.5% ± 6.9%, and the most affected season by positive changes is spring with an average increase of 10% ± 5.9%, the latter mainly located in High Mountain Asia. The results indicated a shift in the snow regime located between the 80 s and 90 s of the previous century, where the period from 1982 to 1999 is characterized by a higher number of areas with significant changes and a higher rate of changes with respect to the period 2000–2020. This quantification can lead to a more accurate evaluation of the impact on water resources for mountainous communities.

## Introduction

Snow cover plays a key role over the Earth surface because of its relationship with the terrestrial climate system, and since it is a hydrological variable very sensitive to climate changes^1–5^. In this context, mountain areas have a very relevant role because of the known amplification effect of climate changes, with cascading effects on downstream areas^6–8^. In fact, since mountains are considered as water towers, changes in the amount, timing and duration of the snow cover extent have several implications on water availability. It is estimated that approximately 15% of the total seasonal snow area above 40° N is in alpine regions of North America, and about 13% in Eurasian^9^. Understanding changes in the snow cover extent in the last 30–40 years can be very relevant to quantify the impact on water resources and to define adaptation strategies for the upcoming decades.

The trends of different snow cover parameters in the last decades over different mountain ranges appear to be quite variable. Smith and Bookhagen^10^ analyzed time series of high-resolution snow water equivalent data over High Mountain Asia from 1986 to 2016, indicating mainly negative trends, while in the areas of Karakorum, Hindu Kush and Kunlun Shan positive trends are found during the winter and summer periods. Regions with positive trends generally correspond to regions of positive glacier mass balances.

The analysis by Pulliainen et al.^9^ revealed that snow mass decreased by 46 gigatons per decade across North America but had a negligible trend across Eurasia: both continents exhibit high regional variability. Positive trends are found in west-central and eastern Siberia and as well in the Praire region in USA/Canada.

The analysis performed on maximum seasonal snow depth in North America also pointed to a robust negative trend in this variable for the period from winter 1960/1961 to winter 2014/2015^11^. Similar decreasing trends were found by using both ground data in the Swiss Alps^12^ and model simulations in Austria^13^. This is in line with the picture drawn by Beniston et al. in the review of the status of the European mountain cryosphere. For the European Alps, a recent study by Matiu et al.^14^ found that over all stations and all months, 87% of the trends were negative and 13% positive, with marked changes in the spring months and at lower elevations, while general decreasing trends at all elevations were identified in the Swiss Alps by Klein et al.^15^. The Fennoscandian mountain areas also showed similar contrasting trends, while mountain areas located in Spain, Romania, Croatia and Bulgaria mainly suffered from snow cover reduction.

To monitor snow cover changes and assess the impact on water resources and climate, long time series (> 30 years) of consistent data are needed^9,16^. Most studies focused on the use of point measurements from complex terrain that normally provide a specific representativeness, depending on the area where the stations are located. Consequently, the interpolation of ground measurements, especially those with weak data coverage in complex orography, may require adaptive interpolation methods^14^. The use of modeled and/or reanalysis data can have some advantages with respect to ground data and satellite observations, being more consistent in time and space^17^. However, the coarse spatial resolution of the modeled and/or reanalysis data sets resulted in difficulty to well represent the spatial variability of mountain processes^18–20^. In this context, downscaling approaches can be used to improve the low resolution modeled data through the use of high resolution data derived from satellite imagery^21,22^.

Various techniques for downscaling snow cover parameters can be found in the literature. More recent works have started adopting methods based on machine learning, which can easily deal with the non-linearity among variables and large data sets. Among the different machine learning approaches, Artificial Neural Networks (ANNs) have been widely adopted^19,20,23^.

As above mentioned, there is a need to assess the status of snow cover parameters in mountains at global level for the long period (30–40 years). However, different studies focus on data that do not have sufficient ground resolution to capture mountain variability, or exploit ground data that mainly represent local conditions. Moreover, the longest time series of satellite images with sufficient ground resolution such as MODIS is limited to around 21 years, thus not allowing assessment of long term trends.

In this framework, the main objectives of this paper are: (1) to generate the first consistent and accurate data records of snow cover area (SCA) and snow cover duration (SCD) over global mountain areas from 1982 to 2020, by downscaling modeled data with satellite records through a machine learning approach; (2) to analyze trends in snow cover extent and snow cover duration throughout different seasons: September–October-November (SON), December-January–February (DJF), March–April-May (MAM) and June-July–August (JJA), as well as different time-frames (1982–2020, 1982–1999, 1990–2010, 2000–2020).

## Results

### Snow cover extent and duration trends from 1982 to 2020

**Figure 1:**
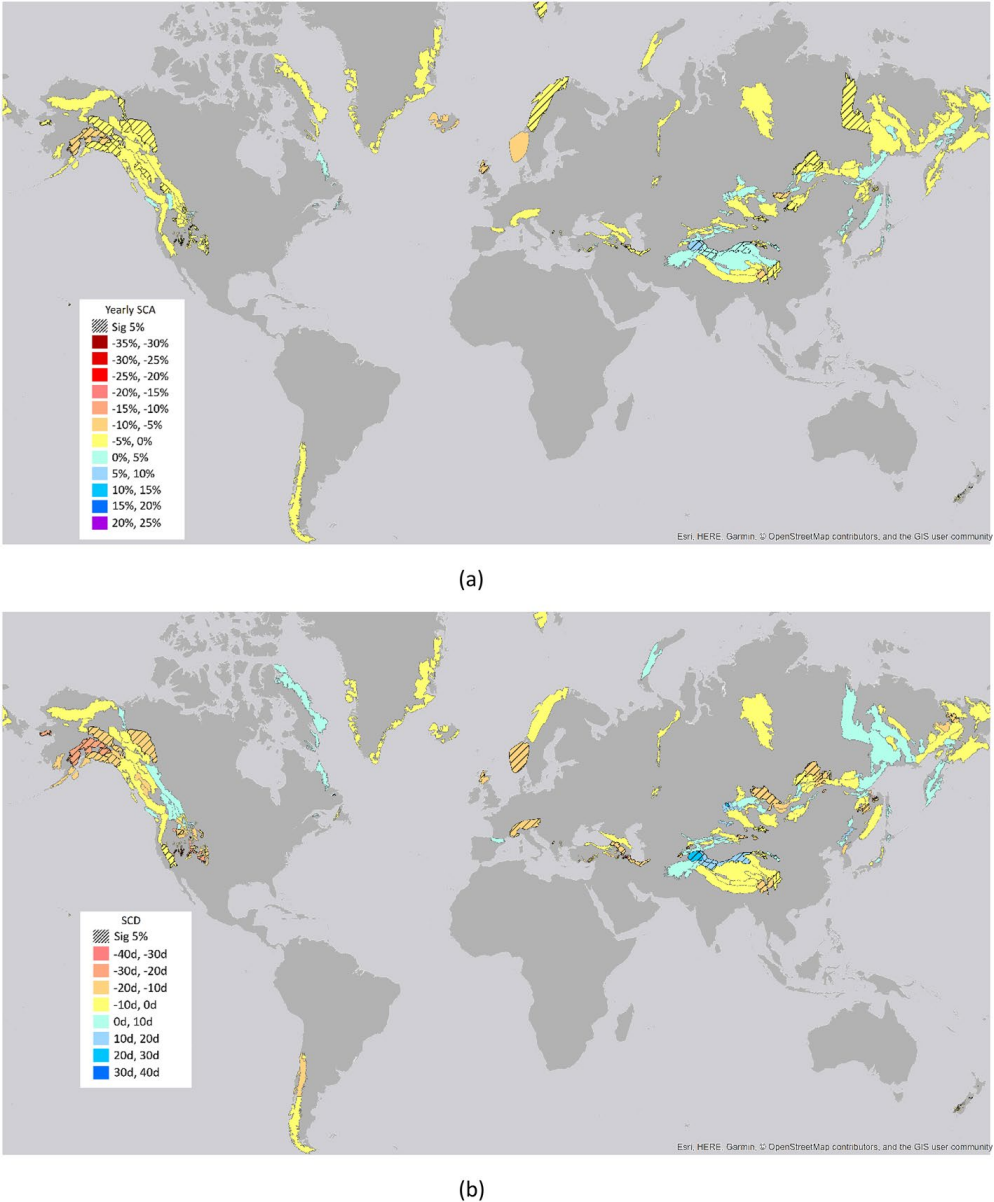
Global trends over 1982–2020 for (**a**) SCA and (**b**) SCD parameters. The figure shows the results of the Mann–Kendall statistics over mountain areas. The underlined areas show significant trends at 5% level (the maps were created by the author using the software ARCGIS v.10.1, www.esri.com).

**Figure 2:**
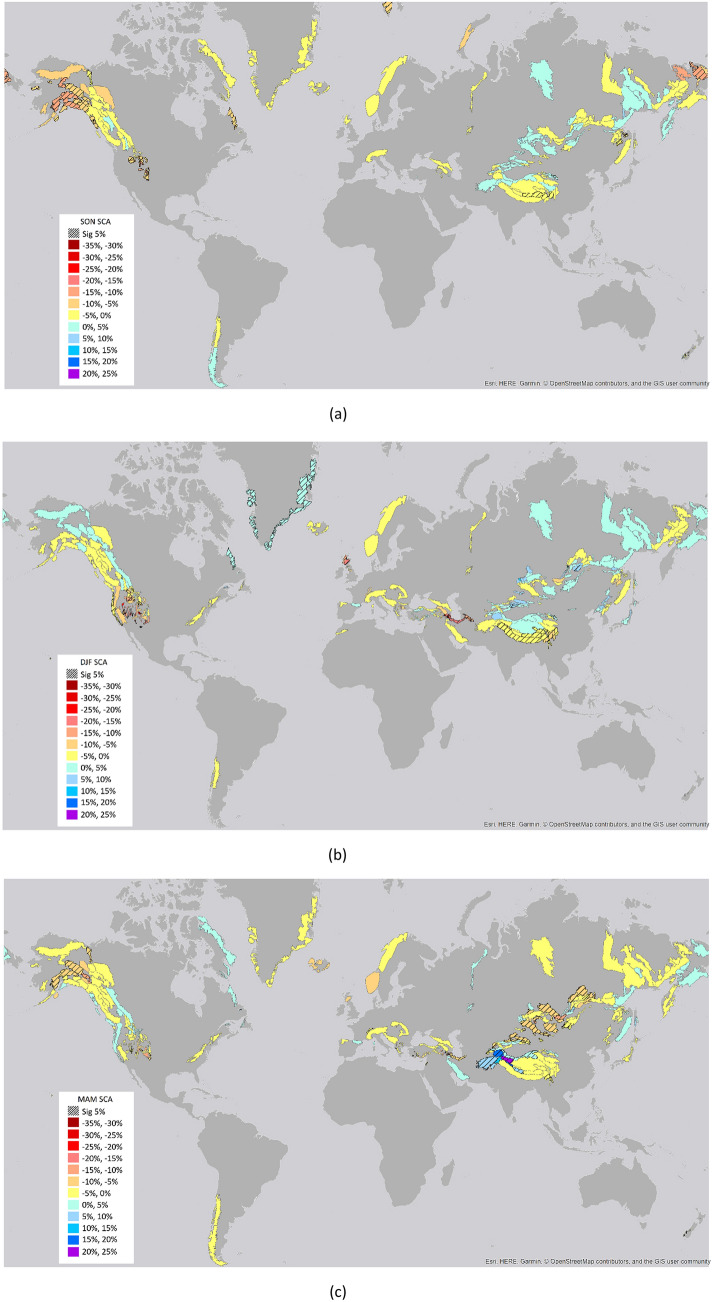

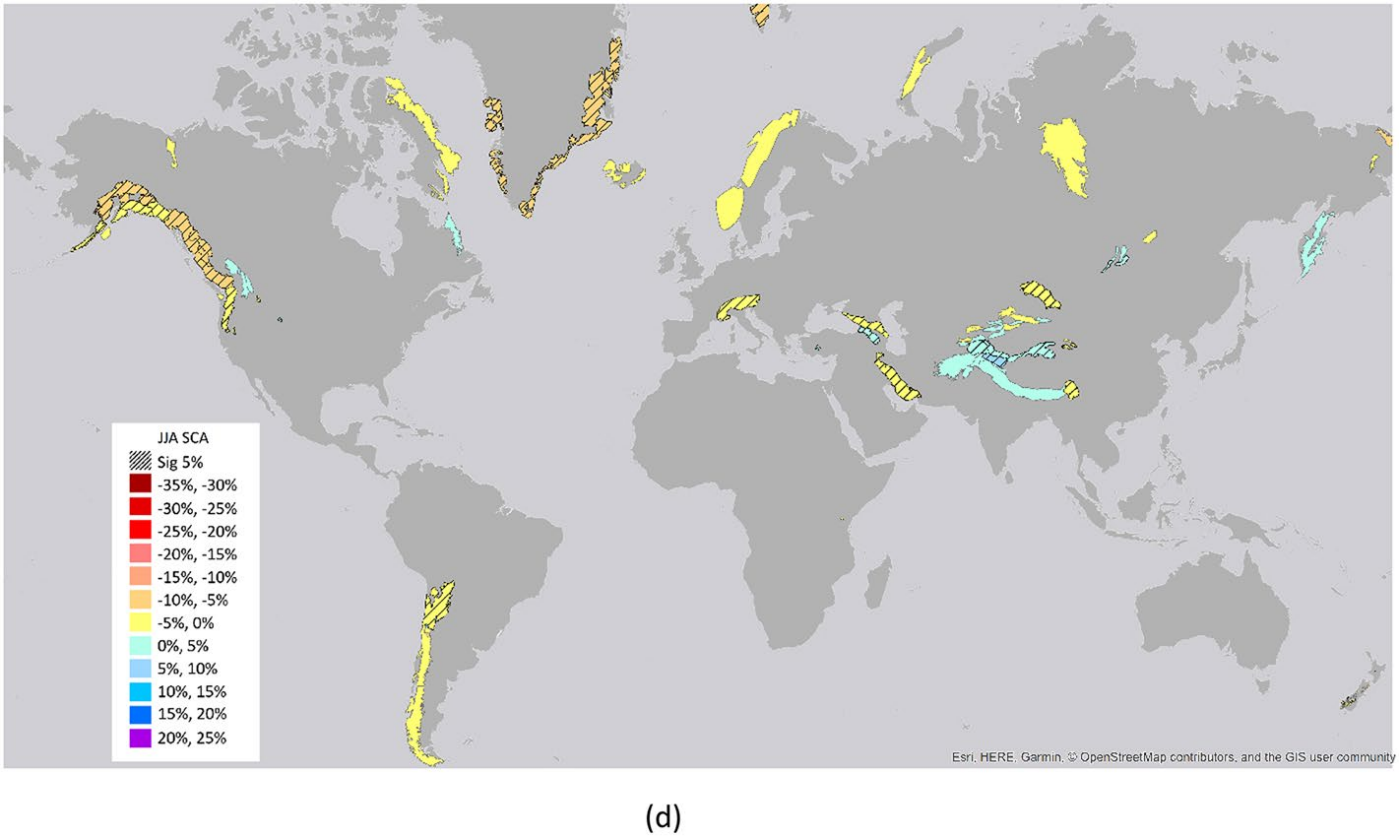
Global trends over 1982–2020 for SCA values in the different seasons: (**a**) September–October–November (SON), (**b**) December–January–February (DJF), (**c**) March–April–May (MAM), (**d**) June–July–August (JJA). The underlined areas show significant trends at 5% level (the maps were created by the author using the software ARCGIS v.10.1, www.esri.com).

Aggregate trends over global mountain areas are slightly negative with an averaged snow cover extent reduction of 3.6% ± 2.7%, and a snow cover duration decrease of 15.6 days ± 11.6 days in 38 years, including only trends with p < 0.05, and areas with at least a yearly average of SCA > 10% and a yearly average of SCD > 20 days (Figs. 1, 2). While the aggregate trends appear to be small, a high dynamic in the different mountain ranges throughout the seasons and timeframes was found^24^. All results were verified with a dedicated uncertainty analysis as detailed in the methodology section.

SCA and SCD significant changes are mainly located in the Northern Hemisphere, more specifically in Canada and Eurasia (Fig. 1a,b). For SCA, the negative changes are up to − 8.2% (− 13.5%, − 1.4%) in the Grampian Mountains in England, and the positive changes are up to 5.1% (1.0%, 8.9%) in the Pamir Mountains (Afghanistan, Pakistan, China, Kyrgyzstan) (Fig. 1a). For SCD, the negative changes can reach − 39.3 days (− 63.2 days, − 16.6 days), detected in Toiyabe Range (USA), while the positive changes reach up to 27.4 days (6.8 days, 42.6 days) in the Pamir Mountains (Afghanistan, Pakistan, China, Kyrgyzstan). Similar patterns can be recognized in Pulliainen et al.^9^, such as snow mass decrease in Canada and Europe, and increase across large portions of Siberia (especially in the eastern part) and in some coastal regions (Arctic Ocean and Japan Sea coasts) (Fig. 1b). Pullianen et al.^9^ analyzed trends over the 39-year satellite record over the Northern Hemisphere, even though they excluded mountain areas due to the low ground resolution of the GlobSnow products (25 km × 25 km) and limited the analysis to March.

In Fig. 1a, it is worth underlining the different behavior of SCA trends between Northern and Southern Norway. The two trends are very close to each other. The SCA trend in Southern Norway is − 6.37% and it is significant at 10% with a Z-score of 1.89. The one in northern Norway has a trend of − 4.69% with a Z-score of 2.04 and it is significant at 5%. The latter shows less variability in data with respect to Southern Norway and for this reason the score is found significant at 5% level.

Western Canada and USA showed a high variability always dominated by snow cover decline (Fig. 1a). In general, a delayed start of the season showed to be stronger than an anticipated melting season (Fig. 2a,c), as detected also by Hori et al.^25^. Declining snow cover was observed by several authors such as Mote et al.^26^, who detected over the period 1955–2018 a strong snowpack decline for 90% of the snow monitoring sites, and Scott and Kaiser 2004^27^, who used ground stations from 1948/49 to 2000/2001 in the western USA, especially in the Pacific Northwest areas. On the other side, Pederson et al. 2013^28^ analyzed April 1st snow water equivalent (SWE) in the western USA and found that since 1980 negative changes in the northern and southern Rocky Mountains are associated with springtime warming.

**Figure 3:**
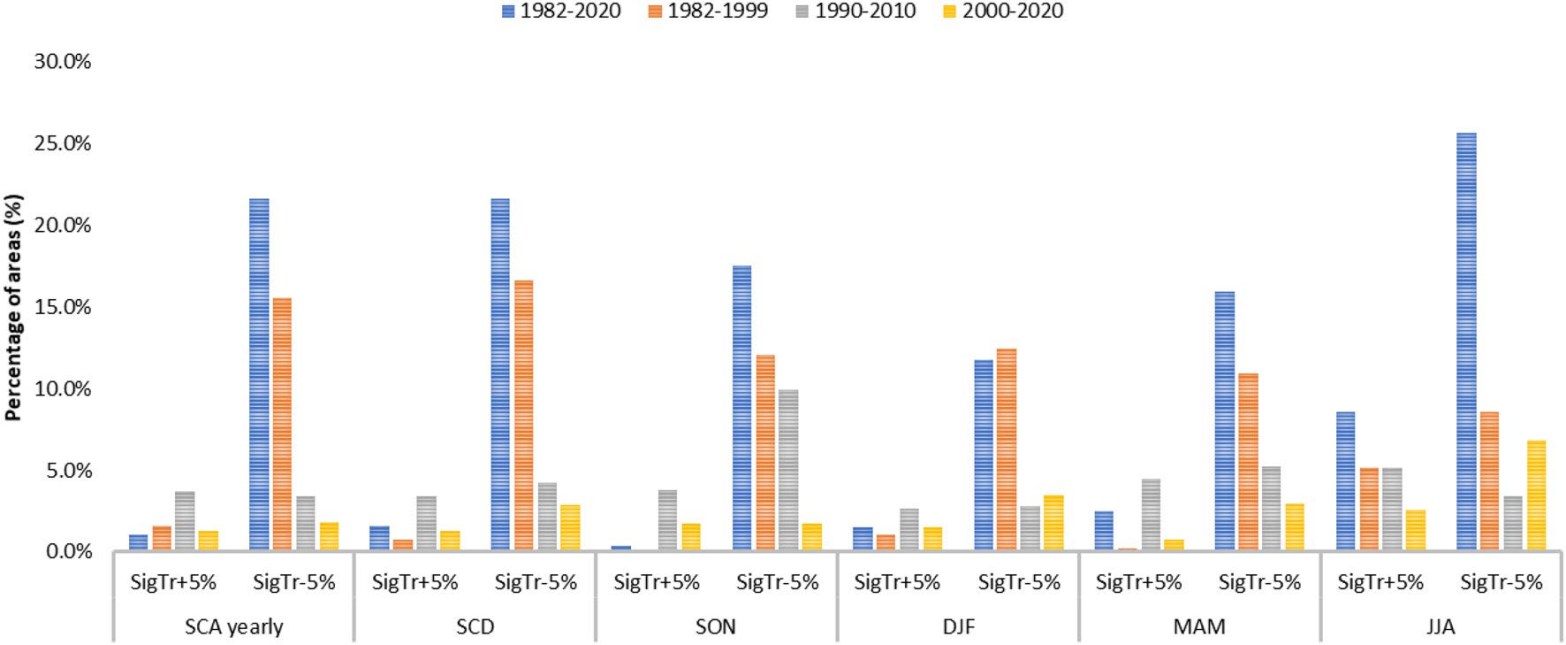
Percentage of areas with significant trends. Percentage of areas with respect to the total number of areas with significant positive and negative trends for the long-term period (1982–2020) and the three sub-periods analyzed (1982–2000, 1990–2010, 2000–2020).

**Figure 4:**
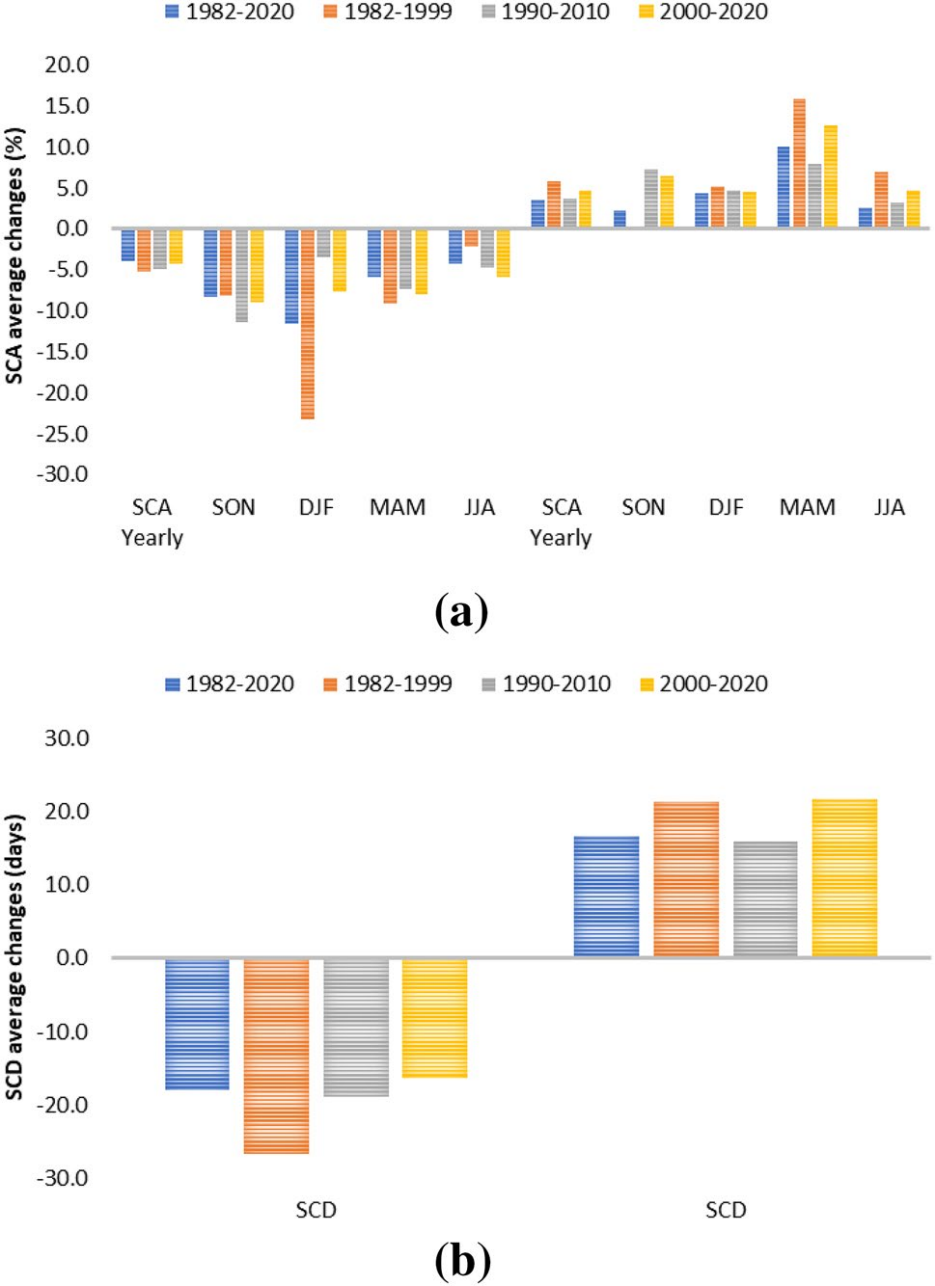
Average magnitude of positive and negative changes for (**a**) SCA at year level and in different seasons, (**b**) for SCD. The average is calculated over the areas with a significant trend at 5% level.

In the different seasons, the largest area with negative changes is detected in SON and JJA, while in DJF the highest magnitude of negative changes is detected with an average SCA decrease of − 11.5% ± 6.9%. For the positive changes, the largest area is in MAM and JJA and the highest magnitude of changes is in MAM with an average SCA increase of 10.0% ± 5.9% (Figs. 3, 4). This high positive value is mainly related to the area of High Mountain Asia (e.g. Kunlun Shan, Karakorum, Malakand Range, Pamir Mountains, Hindu Kush). Results over this area indicated an overall slightly negative trend for the whole period 1987–2016, while the only region which maintains positive trends through the full year and in each seasonal slice is the Karakoram-Kunlun Shan region^10^. These positive trends are visible through spring (March–April-May) in the Pamir-Karakoram-Kunlun Shan region, and in the Tien Shan region with strong positive winters (December–January–February), as detected by the current analysis^29^.

Considering that glacier variability can also impact the detection of snow changes, the areal extent of glaciers for each area was evaluated by using the Randolph Glacier Inventory^30^. In areas with at least 10% glacier coverage, the trends for yearly SCA and SCD were all negative apart in Karakoroum^10^. Also in MAM period Karakorum and Malakand Range show positive trends. Areas with positive trends match well with regions of positive glacier mass balance, as presented by Shean et al. (2020)^31^.

### Analysis of different time periods

During the 38 years long analysis, the timing and magnitude of SCA and SCD trends were also investigated by considering periods of 20 years, starting in 1982, 1990, and 2000 respectively. In this case, the analysis for the periods 1982–1999, 1990–2010 and 2000–2020 in terms of percentages of areas with significant positive and negative changes and the average change values are reported in Figs. 3 and 4 respectively. Long-term (38 years) SCA and SCD trends are generally negative; these trends also correspond to a particularly negative period of short-term trends starting in the late 1980s^2^. Trends based on 20 years periods show positive signals starting in the 1990s^10,32^.

Analyzing the three different periods in comparison with the long-term trends, for SCA around 21% of the total areas have significant negative long-term trends, as well as negative trends in one or two of the other periods, while around half of them keep negative trends only for the period 1982–1999. Four main areas (Lenglong Ling, Pamir Mountains, Karakorum, Kunlun Shan) show long-term positive trends and maintain them for one of the other periods. Due to the strong correlations between SCA and SCD, similar results are obtained for SCD. Among the seasons, SON and DJF follow similar trends of SCA and SCD, while for MAM it is worthwhile mentioning five areas (Iri Dagh, Küh haye Sabalan, Küh-e Bozqush, Küh-e Sahand located in Iran and Türgen Uul in Mongolia) that show negative trends for both the long term and all the other analyzed periods. Pamir Mountains show positive trends for the long term and for the periods 1982–1999 and 2000–2020. For JJA, three areas (European Alps, Zagros Mountains and Greater Caucasus) show negative trends for the long term and for the periods 1982–1999 and 2000–2020. Several studies highlighted this regime shift with high snow amounts in the 1960s and 1970s and negative anomalies in the 1980s and 1990s, in France, Switzerland, Italy, and the western and southern part of Austria, and a recovery afterwards^14,33–36^.

**Figure 5:**
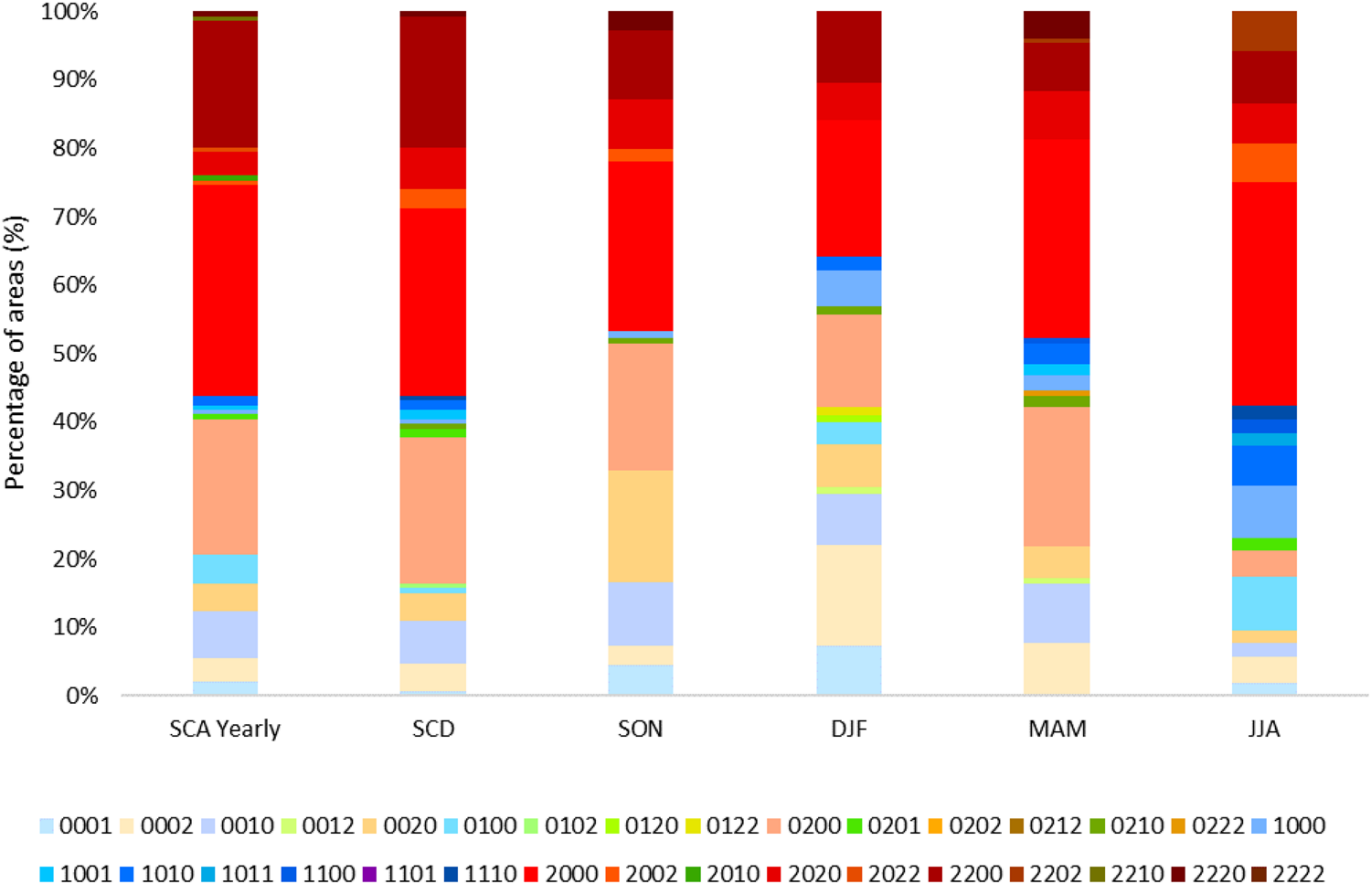
Distributions of the areas (in % with respect to the total number of areas) for trends in different periods. In the legend, the digit of thousands indicates significant trends in 1982–2020, the digit of hundreds in 1982–2000, the digit of tens in 1990–2010, the single digit in 2000–2020. The value “2” indicates negative significant trends and the value “1” represents positive significant trends. For example: “0002” indicates a negative trend for the last period 2000–2020 and no significant trends in the other analyzed period. “2000” indicates a negative significant trend for the period 1982–2020 and no significant trends in the other analyzed periods. The colors in the figure are the same of Figs. S1 and S2.

The overall behavior of trends for the analyzed long-term period and three 20-year periods are shown in Fig. 5, while the geographical representation is reported in the supplementary material (Figs. S1, S2).

In final remarks it is worthwhile mentioning that some differences in the trend magnitude and direction between the results of the current study and other works can be due mainly to three factors: the type of data used, the related scale and the analyzed time spans. Another main difference is that this study focuses only on mountain areas at global level, while other studies provide results at continental scale or limited to some mountain ranges.

## Discussion

The study shows an overall negative trend over the global mountain areas in the period 1982–2020 and this is consistent with observations on the mountain surface air temperature in areas such as Western North America, European Alps and High Mountain areas, which show a warming rate of 0.3 °C ± 0.2 °C per decade^38^. This indicates a faster increase compared to the global warming rate of 0.2 ± 0.1 °C per decade^37,38^. Local warming rates depend on the season, for example in the European Alps it has been found to be more pronounced in summer and spring^39^, while on Tibetan Plateau warming is stronger in winter^38^. This is confirmed by the findings in this study that indicate significant negative changes for the European Alps during the period June-July–August, while for High Mountain Asia negative significant trends are found in the winter period (December-January–February). Notwithstanding the important role of temperature changes, precipitation is also a critical control on mountain hydrological resources. Precipitation changes are less quantified than temperature changes and are often more heterogeneous even within the same mountain ranges^38^.

While global trends go in the clear direction of a decline for snow parameters, locally several trends emerge and may be related to different drivers.

The presence of negative trends in Canada and USA is still controversial. The reasons for the overall negative trend can be related to different combined factors. Main drivers can be due to warming of the minimum temperature, decrease in winter precipitation, decreases in the ratio of snowfall to total precipitation^27,40^. Thus, increased within-season melting of the snowpack and more precipitation falling as rain could be contributors to the negative trend in maximum snow depth^11^.

In High Mountain Asia there are strong local variations for both the long-term trends and the different sub-periods. In general, the area is divided into two main sub-regions (Figs. 1, 2), the northern part with areas such as Karakorum, Kunlun Shan, Pamir Mountain with positive trends and the southern part with areas such as Mishmi Hills, Patkai Hills, Himalaya with predominant negative trends^10^. These behaviours may be driven by differences in climatic conditions, major weather systems in the region such as the Indian summer monsoon, the Eastern Asia summer monsoon and the winter westerly disturbances. Especially the positive trends can be linked to increase of snowfall related to changes in the winter westerly disturbances^10^. Moreover, other factors such as dust and aerosol melt forcing between regions may play a role in the resulting trends^41,42^.

The results presented here indicate different trends within the 20-year sub-periods considered in the analysis. Specifically, most regions show stronger negative trends in the period 1982–1999 than in the period 2000–2020. This is reflected as well in the decreasing rates of changes both for the positive and negative trends. As indicated by several authors, this can be related to impacts on the cryosphere due to regime shifts in the 80 s and 90 s. Reid et al.^32^ detected the period from 1983 to 1990 as a strong regime shift at planetary scale with impacts on vegetation, cryosphere, frequency of fires and many other parameters. The steady temperature increase is related to a combination of natural and anthropogenic processes. The major volcanic eruption of El Chichon in 1982 was responsible for an estimated cooling of 0.2–0.3 °C per decade, resulting in relatively small global mean temperature negative trends in the early 1980s^43^. By the mid-1980s to the late 1980s, however, recovery from the climatic impacts of the eruption led to a natural warming, that added to the produced anthropogenic warming a rapid increase in global mean temperature to a higher level than before the eruption. As a result, global climate shifted to a warmer state in just a few years, setting in motion a cascade of responses in natural systems^44,45^.

This study provides an overall view of the mountain snow cover extent and duration over the last four decades. These changes can affect several sectors, especially because future projections indicate that snow decline will continue for the twenty first century^46^. A first impact can be related to the increased frequency of natural hazards such as snow avalanches involving wet snow and rain-on-snow flood events. The number of wildfires has increased in Western USA partly due to early snowmelt. On the hydrological side, changes in snow regimes (extent and duration) have direct consequences on river run-off regimes, even though up to now there is limited evidence of impacts on hydropower production due to changes in seasonality and quantity, as observed in different mountain areas worldwide (European Alps, Western Canada and USA and Tropical Andes).

In agriculture, several effects have been observed, going from lower yields due to scarcity of water and dry soils^47^, to the upslope movements of crops because of the rising temperature. The reduced availability of water has also brought to adaptation measures in irrigation with new systems, water conservation measures and infrastructures for water storage. It is also worthwhile mentioning that these adaptation measures in agriculture, as well as in tourism and drinking water supply, have contributed to reducing the impact of changes in the seasonal run-off^38^.

Another relevant aspect is related to mountain ecosystems. In fact, the reduced snow cover extent and duration have endangered some snow-dependent plant and animal species^48–53^.

This study highlights the need and usefulness of long-term records and evenly distributed spatial data in the analysis of snow trends. To this purpose, it highlights the efficiency of machine learning approaches to downscale reanalysis and/or modeled data by using consistent data sets from Earth Observation data. This has a twofold effect: first, to determine improved time series and second, to generate data with a time frame which would not be available using only Earth Observation data sets. The downscaling approach is then needed to generate data which can better represent the peculiarity of mountain areas. In this kind of approaches such as ANNs, it is important to underline the importance of the uncertainties. These were estimated through the bootstrap method, while the mean consistency and trend preservation of the downscaled and original MODIS data were checked with the non- parametric Mann–Whitney and the Kolmogorov–Smirnov tests respectively (see “Methods” section for details). Moreover, the method has the potential to be extended to other cases such as the downscaling of future projections^18,54^.

## Methods

### Satellite data

To generate a full time series tailored to mountain areas, modeled data were downscaled using the full time series of MODIS snow cover maps and MODIS snow cover duration from the hydrological years 2000–2001 to 2019–2020.

Snow Cover Area (SCA) and Snow Cover Duration (SCD) were obtained by the products NASA-Moderate Resolution Imaging Spectroradiometer (MODIS) Daily L3 Global 500 m Grid, Version 6 (MOD10A1.006), available at https://nsidc.org/data/mod10a1. Data were processed for the whole period from February 24th, 2000, up to September 30th, 2020, considering the hydrological year as follows: for the Northern Hemisphere from 1st October to 30th September, for the Southern Hemisphere from 1st April to 31st March^55^. MOD10A1.006 products provide values of the Normalized Differential Snow Index (NDSI) that were transformed into snow cover fraction by using the linear approach proposed by Salomonson and Appel^56^. Snow cover fraction values were transformed into Snow Cover Area (SCA) values by averaging over the different mountain ranges. Data were processed considering yearly and seasonally average for: September–October–November (SON), December–January–February (DJF), March–April–May (MAM) and June–July–August (JJA). The Snow Cover Duration (SCD) values were derived by using the procedure described in Notarnicola 2020^57^. All the processing was carried out using the Google Earth Engine (GEE) platform^58^. MODIS SCA products were extensively validated in different mountain areas in the last two decades, indicating an average accuracy of around 94.2%^59–61^. In mountain areas, the accuracies strongly depend on the illumination conditions and cloudiness. For SCD validation, the comparison with ground data determined the following performances: Mean Absolute Error = 21.1 days, bias = − 3.1 days, R = 0.84. The reader can refer to Notarnicola 2020^57^ for full details on the snow parameter validation.

### Modeled data

The modeled data considered in the work are derived from the simulation of the Famine Early Warning Systems Network (FEWS NET) Land Data Assimilation System (hereafter named FLDAS)^62^. These data contain different land surface parameters obtained from the Noah 3.6.1 model^63^, which simulates the important biogeophysical, hydrological and energy balance processes, thus providing a physical approach to snow modeling. They are produced at monthly basis with a global coverage, and their main characteristic with respect to other models (e.g. GLDAS) is the higher spatial resolution of 0.1° × 0.1° (10 km × 10 km).

The simulation was forced by a combination of the Modern-Era Retrospective analysis for Research and Applications version 2 (MERRA-2) data and Climate Hazards Group InfraRed Precipitation with Station (CHIRPS) 6-hourly rainfall data that have been downscaled using the NASA Land Data Toolkit.

Snow modeling for FEWS NET was developed and originally implemented by the NOAA National Operational Hydrologic Remote Sensing Center (NOHRSC), using a spatially distributed land surface model (LSM) operating within the Land Information System (LIS) software framework version 6. LIS provides a means for high-performance land surface modeling utilizing multiple potential forcings^63^. Additional specifications are available at https://ldas.gsfc.nasa.gov/FLDAS/FLDASspecs.php.

From FLDAS, two main parameters were addressed: snow cover fraction, which can be directly compared with the MODIS SCA products, and snow depth, used to derive SCD maps by considering different thresholds (2 cm, 5 cm, 10 cm).

It is worthwhile mentioning the limitations of the data used in this study. The data used in the downscaling approach can be affected by several uncertainties. MODIS satellite data can offer data with the highest possible ground resolution (500 m) to observe mountain areas with a good level of accuracy. However their estimates can have some limitations, being affected by cloud cover and with limited penetration into the canopy^16,57^. Moreover, these data cover only the period from 2000 onward, thus limiting the attribution of long-term trends. For this purpose, the combination with modeled data offers the possibility to extend the perspective of monitoring to longer time scale. There is a large small-scale variability remaining, due to microclimatic effects not captured in the gridded dataset, and considerable uncertainties can be related to this aspect. For this reason, no altitude analysis was carried out. On the other hand, the results are appropriate to quantify long-term regional trend patterns and impacts on water resources in the specific areas analyzed.

### Study area

The analysis is carried out at global scale in different mountain ranges as delineated in the layer Global Mountain Biodiversity Assessment (GMBA)^64^. The layer reports 1003 mountain ranges by using the place name references that easily allow intercomparison among areas.

A further selection was done on the areas based on the use of satellite and modeled data. Specifically, only those with persistent and seasonal snow cover were considered in the following analysis. They were selected by considering yearly mean snow cover area (SCA) averaged over the period 2000–2001 to 2017–2018 (18 years) larger than 10%, and snow cover duration (SCD) averaged over the same period longer than 20 days. Moreover, same areas were not considered as modeled data provided always null values. After this thresholding, 380 areas remain for yearly SCA and SCD values. For SON period, 291 areas were selected, for DJF 459, for MAM 402 and for JJA 117.

### Comparison of FLDAS snow cover data with MODIS SCA (MOD10A1, 500 m) and SCD (derived parameter) over the period 2000–2018

As the first step in the downscaling procedure, the capacity of the FLDAS snow cover parameter to reproduce the snow seasonal cycle in mountain areas at global level was assessed. FLDAS snow cover mean values were compared with MODIS SCA and SCD as extracted from the product MOD10A1 from the hydrological years 2000–2001 to 2017–2018. This timeframe was selected as these data are the basis for the training and test of the downscaling approach. The remaining years (2018–2019, 2019–2020) are used as blind test to further assess the robustness of the downscaling approach. The products were compared considering the mean at area level at yearly basis, for different elevation belts and season based (SON, DJF, MAM, JJA) and per latitude belts. The following figures of merit were considered: Bias, Mean Absolute Error (MAE), Root Mean Squared Error (RMSE), Pearson correlation coefficient R. Only areas with averaged SCD > 20 days and averaged SCA > 10% were included in the analysis.

Here, the analysis on the elevation was performed considering the mean elevation within each area. The reason of such analysis is related to the ground resolution of the FLDAS snow cover. The results are presented in Table 1.

**Table 1:** Comparison between MODIS SCA and SCD and modeled FLDAS data (*Pearson correlation significant at 5% level). For SCD, different thresholds of Snow Depth (SD) were tested. For each data set, in parenthesis the number of points used in the evaluation is indicated.

Region/area	Bias	MAE	RMSE	R
**Yearly SCA**				
Yearly SCA average (n.6840)	0.0065	0.078	0.097	0.90*
**% tree cover**				
< 20% (n.3636)	0.016	0.071	0.090	0.94*
20–50% (n.2790)	− 0.007	0.081	0.099	0.84*
> 50% (n.414)	0.0135	0.11	0.128	0.59*
**Elevation belts**				
H < 1000 m (n.2034)	− 0.017	0.082	0.099	0.93*
1000 m < H < 2500 m (n.3870)	0.0013	0.072	0.091	0.87*
2500 m < H < 4000 m (n.702)	0.075	0.089	0.110	0.77*
H > 4000 m (n.234)	0.091	0.107	0.120	0.69*
**Latitude**				
< 40°N (n.1710)	0.035	0.073	0.091	0.69*
40°N–50°N (n.2088)	− 0.003	0.076	0.096	0.59*
50°N–60°N (n.1530)	− 0.023	0.085	0.103	0.80*
> 60°N (n.954)	− 0.035	0.060	0.074	0.80*
30–50°S (n.558)	0.105	0.109	0.128	0.70*
**Season SCA**				
SON (n.5238)	0.033	0.096	0.125	0.86*
DJF (n.8262)	0.958	0.153	0.202	0.86*
MAM (n.7236)	0.717	0.110	0.140	0.93*
JJA (n.2106)	0.142	0.157	0.189	0.78*
**Area size (km**^**2**^**)**				
< 500 (n.36)	0.196	0.196	0.213	0.81*
500 < A < 1000 (n.126)	0.085	0.114	0.135	0.74*
1000 < A < 5000 (n.1638)	0.022	0.071	0.087	0.76*
5000 < A < 10,000 (n.1026)	0.006	0.084	0.103	0.81*
10,000 < A < 50,000 (n.2394)	− 0.005	0.074	0.092	0.90*
> 50,000 (n.1620)	− 0.003	0.080	0.100	0.92*
**Snow cover duration (SCD) (d=days)**				
SCD (SD = 2 cm) (n.6840)	− 11.8 d	32.8 d	40.0 d	0.90*
SCD (SD = 5 cm) (n.6840)	9.65 d	33.0 d	40.5 d	0.91*
SCD (SD = 10 cm) (n.6840)	26.0 d	37.2 d	37.2 d	0.91*

The comparison between MODIS and FLDAS for the period 2000–2001 to 2017–2018 indicates that FLDAS can reproduce well the snow behavior throughout the year and as well for both SCA and SCD. For most of the cases, the differences between MODIS and FLDAS are within the limit of the known uncertainties on the MODIS SCA maps^61,65^. Major problems can be found for high percentage of forest cover (> 50%), for higher elevations (> 4000 m) and areas with small size (< 500 km^2^). These uncertainties can be both related to MODIS limitations, such as the detection of snow under forest, and to spatial resolution of FLDAS, which may limit the detection of snow in smaller areas. Regarding the SCD parameter, the threshold of SD = 5 cm was selected for further analysis as a good compromise considering the performances.

### Approach for downscaling based on Artificial Neural Networks (ANNs)

The main aim of the proposed procedure is to downscale the FLDAS data by using MODIS data sets as a reference through an approach based on Artificial Neural Networks (ANNs). This procedure is aimed at improving the modeling of FLDAS specifically over mountain areas by using Earth Observation data with a higher ground resolution and a sufficient length of the time series.

Downscaling approaches constitute empirical methods to derive relationships between large-scale variables (predictors) and observed local variables (predictands). As predictands, several variables can be used related to high resolution imagery and/or geomorphology features, as well as both station and gridded data sets^21,22,66^.

Among the different approaches, ANNs were selected because of their versatility, capability to deal with different types of data and previous experiences^19,23,67,68^.

ANN structure includes many parallel computing units (named neurons) arranged in different layers and linked through adjustable connections (named weights). The weights are the adaptive parts of the set of nonlinear basis functions which compose the ANNs, and are set initially in a random way. Afterward they are adapted to the specific problem by the training process carried out on input/output cases available in the training data set. The training set is a subset of the entire data set which is normally divided into training, validation and test data sets. The training process is an iterative process with the aim to minimize a cost function, for example the average squared error between the network outputs and the targets^69^. ANNs have been used for several purposes: among them the estimation of biophysical parameters^67,70,71^, pattern recognition and downscaling approaches^20^. In this work, a classical feed-forward network with error backpropagation algorithm was adopted.

As a first step, ANNs were used by testing different input configurations and model structure (in terms of number of layers and neurons). For the training, test and validation, all the hydrological years from 2000–2001 to 2017–2018 were used. The total data sets used had the following number: 6840 for yearly SCA and SCD, 5238 for SON, 8262 for DJF, 7236 for MAM and 2106 for JJA. An AAN model was derived separately for the yearly SCA, SCD and the four periods, SON, DJF, MAM and JJA. The subdivision among training, test and validation was 60%, 20%, 20%. As a blind test, data from hydrological years 2018–2019 and 2019–2020 were used, to further assess the capability and robustness of the networks to correct the bias between FLDAS data and MODIS in unforeseen data samples.

The potential input features were selected based on their likely physical relationship to snow cover extent and snow cover duration. The considered input features were of two types: fixed variables which can describe the specificity of the location, such as the mean elevation, the latitude, the average of the reference period of snow cover extent and snow cover duration^21,72,73^. As indicated in Table 1, the comparison between MODIS and FLDAS data shows sensitivity with respect to these parameters. Along with these fixed variables, the dynamic component was also considered by taking into account the changes from location to location and from one year to the other.

Temperature was considered a key input feature, as it regulates whether a location will receive rain or snow and also determines the intensity of snowfall. The other key feature directly related to snow variability was the snowfall values. Both parameters were derived from MERRA2 reanalysis data.

The results indicate that the best performances are obtained when using both fixed parameters and meteorological variables, together with a network of 2 layers with 25 neurons. When considering both fixed parameters and meteorological variables, the relationship between MODIS and FLDAS notably improves. After this analysis, the considered input variables were the following:

- Averaged SCA and SCD (avSCA, avSCD) for the hydrological years from 2000–2001 to 2017–2018
- Temperature (T) and precipitation (P)
- Mean elevation over the area (meanH)
- Centre latitude of the area (L)
- Tree cover in % (TC).

**Figure 6:**
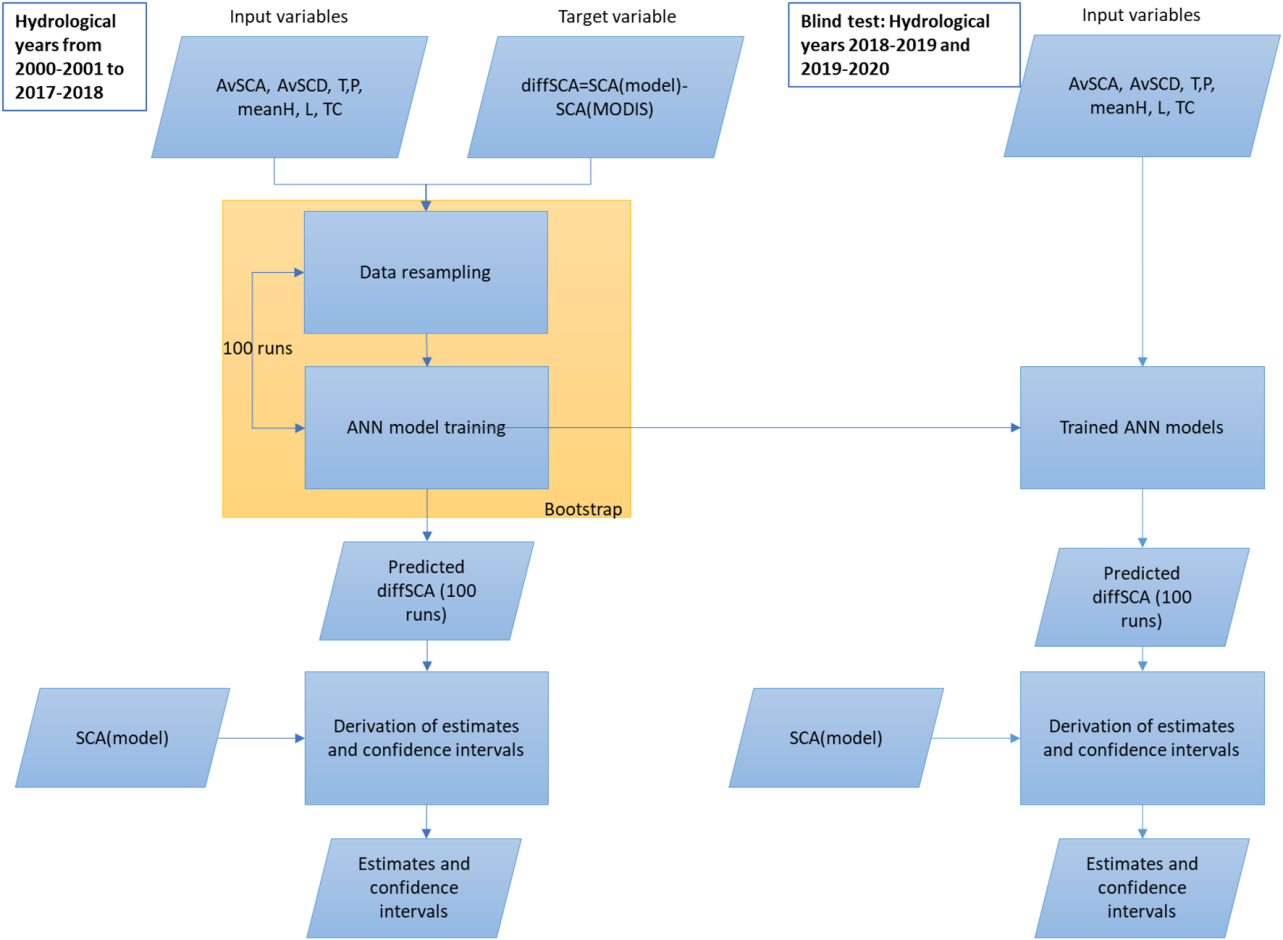
Flowchart of the overall procedure. It is shown the case where the target variable is the yearly SCA. The same applies for the other considered target variables that is SCD, SON SCA, DJF SCA, MAM SCA, JJA SCA.

Regarding the configuration, several tests were run, considering from one to four internal layers and from 10 to 50 neurons for each layer. The results indicate that beyond 2 layers the performances worsen, whereas increasing the number of neurons the performances remain quite stable. These results are shown in the supplementary material (Fig. S3). The flowchart of the procedure is shown in Fig. 6.

The ANN approach was applied to the baseline period from 2000–2001 and 2017–2018, where both FLDAS model simulations and MODIS estimates were available. In this period, the years 2018–2019 and 2019–2020 were used as blind test. After that, the trained ANN models were applied to the period 1982–1999.

It is worthwhile mentioning that the target variable was considered the difference between MODIS estimates and FLDAS model. For example in the case of SCA, it was diffSCA = SCA(model) − SCA(MODIS). In this way, the ANN model after the training was able to predict the difference between the two sources of information. To obtain the original target, it is sufficient to add the estimated difference to the corresponding value from FLDAS model. This selection of the target variable is relevant especially when the trained networks were applied to the period 1982–1999, where no MODIS estimates are available.

To quantify the model uncertainty introduced by the ANN empirical regression models, the confidence intervals of the regression error were estimated by using the bootstrap method, based on a resampling technique^74–77^. This approach is quite efficient, simple to implement and provides accurate uncertainty estimates. Moreover, it requires no prior knowledge about the distribution function of the underlying population (e.g. normality), being a distribution-free inference method^74,75^.

The bootstrap approach was based on a resampling with replacement of the original data set. From each bootstrap data set, a bootstrapped regression model is built, and the model outputs are computed. Different bootstrap data sets give rise to a distribution, and a probability density function (PDF) of the model output can be built. Thus, the model uncertainty of the estimates provided by the ANNs can be quantified in terms of confidence intervals^78^. However, the computational cost could be high when the training data set and the number of parameters in the regression model are large^79^. Several tests were run in order to evaluate the adequate number of bootstrap runs as a compromise between stability of the results and computation burden. Considering 50, 100 and 500 runs, it was found that between 100 and 500 runs the results on the confidence interval remain stable, and for this reason the number of bootstrapped data sets was set to 100. To train 100 networks, the processing time was around 3 h in a normal PC. After the processing, the mean estimates with the associated 95% confidence intervals were calculated.

As the last point, it is worthwhile underlining that the downscaling approach was applied on an area-based average, considering that downscaling at pixel basis would have required a more demanding process, including specific modeling of fine scale processes. In fact, the coarse resolution of the initial modeled data and the scales at which they are conceived need to be considered in the downscaling approaches^80^.

### Uncertainty evaluation: confidence interval from bootstrap, blind validation, hypothesis test

The source of uncertainties can be related to the uncertainties in the reference data sets, in the model parametrization, as well as in the proposed downscaling approach. To have confidence on the FLDAS downscaled outputs with MODIS datasets, one important part is that the downscaled outputs can represent the current state of the snow changes reasonably well. This means that the downscaling output ability to represent the MODIS data in the baseline period (2000–2001 to 2017–2018) is important to have reasonable confidence on the reliability of the SCA and SCD change, computed over the whole period 1982–2020. The aim of the uncertainty analysis is, therefore, to evaluate the performance of the downscaling method in reproducing the mean value and variability of observed variables for the baseline period^68,81^.

Three complementary methods were employed to analyze the uncertainty of the output of the statistical downscaling model. First the downscaled results were evaluated on the data of 2018–2019 and 2019–2020 as a further validation to check the robustness of the approach in a new data set. In this first part of the validation, the reference data are always the MODIS derived parameters.

Second, it was important to quantify the impact of the downscaling accuracy on the consideration of the snow cover and parameter change analysis. The main questions are: how reliable are the estimated trends on the downscaled analysis? How do they compare with the MODIS trends in the period where both are available? To answer these questions, the following procedure was set. Once the trends were detected and the related changes derived by using the Mann–Kendall approach and the Sen’s slope respectively, two hypothesis tests were run: non- parametric Mann–Whitney test to check the mean consistency of the downscaled and original MODIS trends, and Kolmogorov–Smirnov test for trend preservation on Mann–Kendall results^68,82^. The data which had significant trends but not satisfying the hypothesis tests were excluded from the final analysis.

Third, if the values of the changes detected by the Sen’s slope were smaller than the confidence intervals obtained with the bootstrap procedure, i.e. the associated uncertainties derived from the ANN procedure, these data were excluded from the final analysis.

These three steps are detailed in the next section.

### Downscaling results from application of ANN approach

#### Validation with MODIS data

Here the performances on the ANN results are presented in two ways. The results on the overall data sets obtained by using the bootstrap procedure imply a resampling of the input data and then a cross-validation is carried out in the procedure. To further assess the robustness of the procedure, the models were applied to the years 2018–2019 and 2019–2020, considering this as a full blind test as these data were never used in the derivation of the ANN models.

The results in Table 3, when compared with those in Table 1, clearly indicate the improvement achieved in the downscaling procedure. The downscaling approach specifically contributed to improve the values of the Bias, MAE and RMSE. When comparing these values in Tables 1 and 2, overall it was found a reduction of root mean squared error of 76%, of mean absolute error of 79%, of bias of 96% and improvement of the correlation coefficient of 12%. The results on the blind test sets (2018–2019, 2019–2020) are consistent with the ANN test data, thus indicating a good capability to reproduce the original MODIS data also when the ANN models are applied to completely unforeseen data. For sake of comparison in Table 3, the performances on the downscaled data are subdivided in different categories as in Table 1. For each category, a clear improvement is found. More in details, the bias values are always below 1%, apart for the JJA months where it is at 8%. The MAE values range between 1.3% and 4.5% and the RMSE values between 2.5% and 6%. All these values are within the limits of the accuracy found for MODIS SCA and SCD values^57,61,65^.

**Table 2:** Performances of the ANNs for yearly SCA, SCD, SON SCA, DJF SCA, MAM SCA, JJA SCA. For each data set, in parenthesis the number of points used in the evaluation is indicated. Values in bold are significant at 5% level.

Parameters	Years from 2000/2001 to 2017/2018				Parameters	Years 2018/2019–2019/2020 (blind test)			
	Bias	MAE	RMSE	R		Bias	MAE	RMSE	R
Yearly SCA (n.6840)	− 0.00004	0.024	0.031	**0.985**	Yearly SCA (n.760)	− 0.0004	0.0342	0.0427	**0.97**
DJF SCA (n.8262)	− 0.00009	0.045	0.063	**0.97**	DJF SCA (n.918)	− 0.004	0.053	0.074	**0.96**
MAM SCA (n.7236)	− 0.0002	0.03	0.04	**0.98**	MAM SCA (n.1206)	0.001	0.04	0.06	**0.97**
SON SCA (n.5236)	− 0.00012	0.04	0.05	**0.96**	SON SCA (n.582)	− 0.002	0.05	0.06	**0.94**
JJA SCA (n.2106)	0.08	0.03	0.04	**0.97**	JJA SCA (n.351)	− 0.02	0.04	0.06	**0.94**
SCD (n.6840) (in days)	− 0.008	7.41	10.11	**0.98**	SCD (n.760) (in days)	0.82	9.42	13	**0.98**

**Table 3:** Comparison between MODIS SCA and SCD and modeled FLDAS data after the downscaling procedure (*Pearson correlation significant at 5% level). For each data set, in parenthesis the number of points used in the evaluation is indicated.

Region/area	Bias	MAE	RMSE	R
**Yearly SCA**				
Yearly SCA average (n.6840)	− 0.00004	0.024	0.031	0.98*
**% tree cover**				
< 20% (n.3636)	0.00001	0.025	0.033	0.99*
20–50% (n.2790)	− 0.00017	0.023	0.030	0.98*
> 50% (n.414)	0.0004	0.019	0.025	0.95*
**Elevation belts**				
H < 1000 m (n.2034)	0.00017	0.025	0.033	0.99*
1000 m < H < 2500 m (n.3870)	− 0.0003	0.024	0.031	0.98*
2500 m < H < 4000 m (n.702)	0.0005	0.023	0.029	0.96*
H > 4000 m (n.234)	0.0006	0.027	0.037	0.91*
**Latitude**				
< 40°N (n.1710)	0.0006	0.022	0.028	0.95*
40°N–50°N (n.2088)	− 0.0006	0.022	0.029	0.93*
50°N–60°N (n.1530)	− 0.00004	0.026	0.033	0.96*
> 60°N (n.954)	− 0.00001	0.030	0.039	0.93*
30–50°S (n.558)	− 0.00025	0.022	0.030	0.94*
**Season SCA**				
SON (n.5238)	− 0.00012	0.040	0.050	0.96*
DJF (n.8262)	− 0.00009	0.045	0.063	0.97*
MAM (n.7236)	− 0.0002	0.030	0.040	0.98*
JJA (n.2106)	0.08	0.030	0.040	0.97*
**Area size (km**^**2**^**)**				
< 500 (n.36)	0.0013	0.013	0.018	0.98*
500 < A < 1000 (n.126)	0.0018	0.020	0.026	0.97*
1000 < A < 5000 (n.1638)	− 0.0004	0.025	0.032	0.94*
5000 < A < 10,000 (n.1026)	0.0003	0.023	0.031	0.97*
10,000 < A < 50,000 (n.2394)	− 0.0005	0.024	0.031	0.98*
> 50,000 (n.1620)	0.0007	0.024	0.032	0.98*
**Snow cover duration (SCD) (d=days)**				
SCD (SD = 5 cm) (n.6840)	− 0.008 d	7.41 d	10.11 d	0.98*

### Statistical analysis

#### Mann–Kendall and Sen’s slope

The presence of a monotonic increasing or decreasing trend in time was detected by using the non-parametric Mann–Kendall (M–K) statistics, for its capability to deal with the presence of outliers in comparison to Pearson correlation^83^. Then, the slope (change per unit time) of the linear regression was estimated by using a simple non-parametric procedure developed by Sen (1968)^84^ together with a 100 (1- α) % two-sided confidence interval. Finally, considering the number of years, the slope was transformed into the total change value for each of the analyzed variables^85^.

#### Hypothesis tests

One important part was the evaluation of the consistency of the downscaled data with respect to the MODIS data and the capability to reproduce the mean values and the trend^81^.

First, the Wilcoxon-Mann-Withney (W-M-W) test was used to assess whether the two samples of data (downscaled and MODIS data set) are likely to derive from the same population. It is a non-parametric test and thus can be applied without any assumption on the data distribution. This test was applied to the downscaled and MODIS data for the period 2000–2020.

Moreover, because of the unavoidable discrepancies between the downscaled data and the original MODIS values, the resulting trends may be different^82,86,87^. In order to see if the trends of the downscaled data preserve the ones of the MODIS data, the Kolmogorov-Smirnoff approach was used. This approach is used to assess whether two samples come from the same distribution. In this case it was applied to the trend estimated by the M–K approach on the downscaled data, and compared with those derived from the MODIS images. As reported in Table 4, for all the parameters the trends were preserved with significance at 5% level.

**Table 4:** Statistical analysis for the different parameters: N° area-total number of areas analyzed; Sig. M–K trend: the areas with significant trends at 5% level for Mann–Kendall (M–K); Sig. W-M-W test: the areas found with no significant difference for the Wilcoxon-Mann-Withney (W-M-W) statistics; Sig. Changes > CI-Bootstrap: areas whose changes are larger than the uncertainties associated with the downscaling procedure and defined with the Confidence Interval of the bootstrap approach; K-S trend preservation: check for the trend preservation with Kolmogorov–Smirnov (K-S) at 5% significance level.

Parameter	N° area	Sig. M–K trend	Sig.W-M-W test	Sig Changes > CI-bootstrap	K-S trend preservation
Yearly SCA	380	89	86	86	yes
SCD	380	88	88	88	yes
SON SCA	291	53	53	52	yes
DJF SCA	459	65	63	61	yes
MAM SCA	402	77	76	74	yes
JJA SCA	117	42	41	40	yes

#### Selection of changes based on the confidence interval

As the last step, the changes detected by the trend analysis (M–K and Sen’s slope) were compared with the uncertainties associated with downscaling procedure through the confidence interval obtained by the bootstrap approach. The changes smaller than the confidence interval were discarded and not considered in the further analysis.

The results of the application of the previous tests are summarized in Table 4. All the analyses presented in this work are based on the data which satisfy all these tests.

## Supplementary Information

Supplementary Information.

## Data Availability

The data used to derive the time series of snow parameters presented and analyzed in this paper can be available upon request to the author.
