# Supplementary material for: Overall negative trends for snow cover extent and duration in global mountain regions over 1982–2020
Source: Sci Rep. 2022 Aug 12;12:13731. doi: 10.1038/s41598-022-16743-w (PMC9374742; doi:10.1038/s41598-022-16743-w)
Supplement: Supplementary file 1 — Supplementary Information. [file 41598_2022_16743_MOESM1_ESM.docx]

**Supplementary figures**


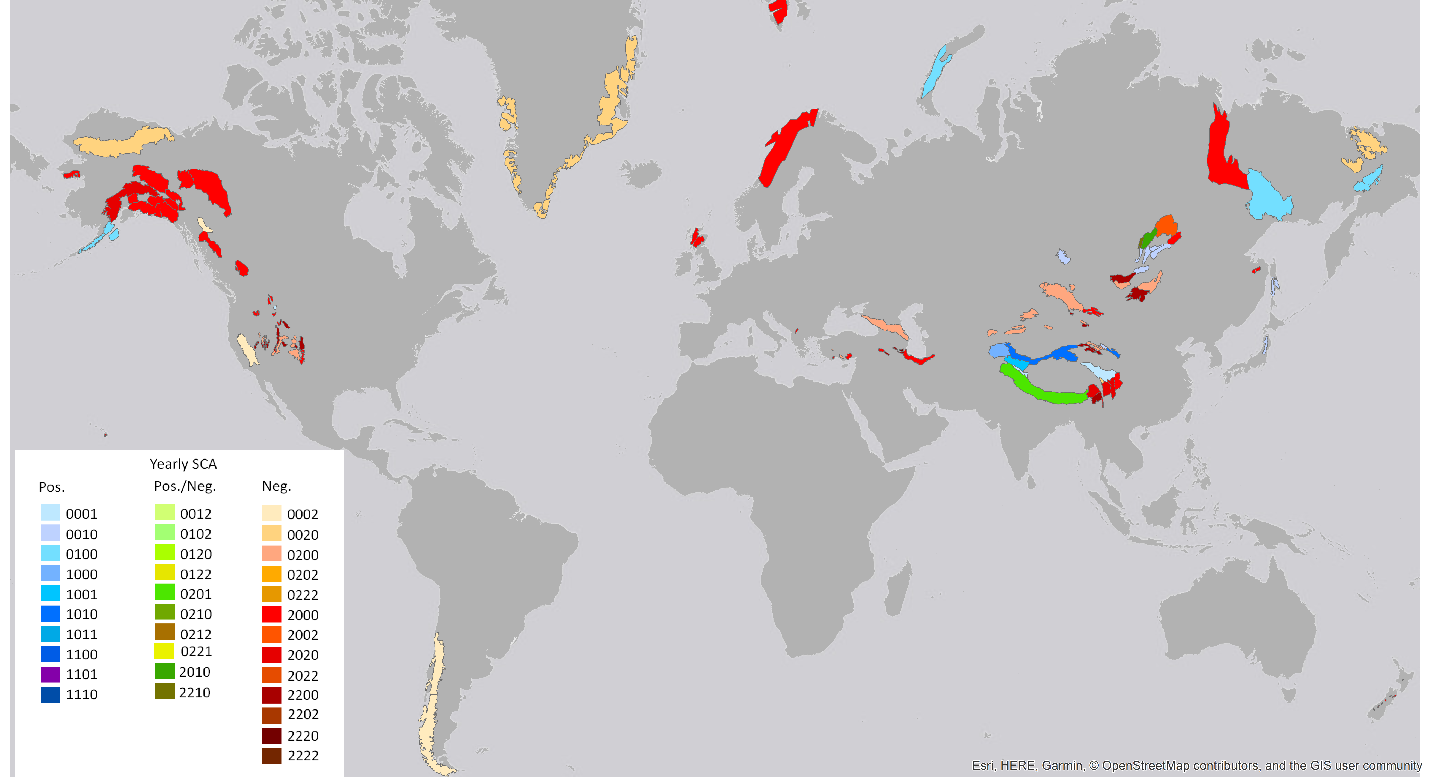


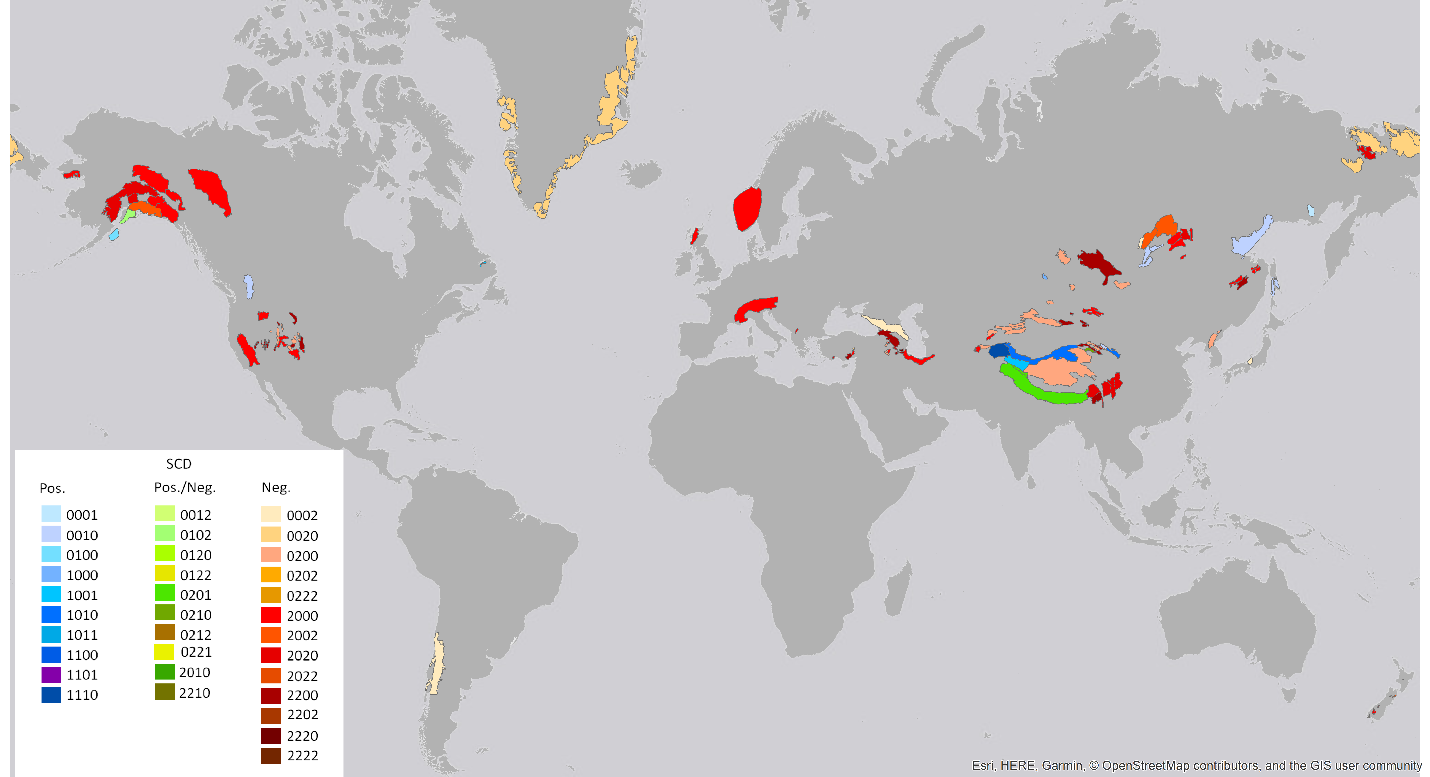


**Figure 1S:** Behaviour of the mountain areas for trends in SCA and SCD in different periods. The four digits in the legend represent the four analyzed time spans: the digit of thousands indicates significant trends in 1982-2020, the digit of hundreds in 1982-2000, the digit of tens in in 1990-2010, the single digit in 2000-2020. The value “2” indicates negative significant trends and the value “1” represents positive significant trends. For example: “0002” indicates a negative trend for the last period 2000-2020 and no significant trends in the other analyzed period. “2000” indicates a negative significant trend for the period 1982-2020 and no significant trends in the other analyzed periods (the maps were created by the author using the software ARCGIS v.10.1, www.esri.com).


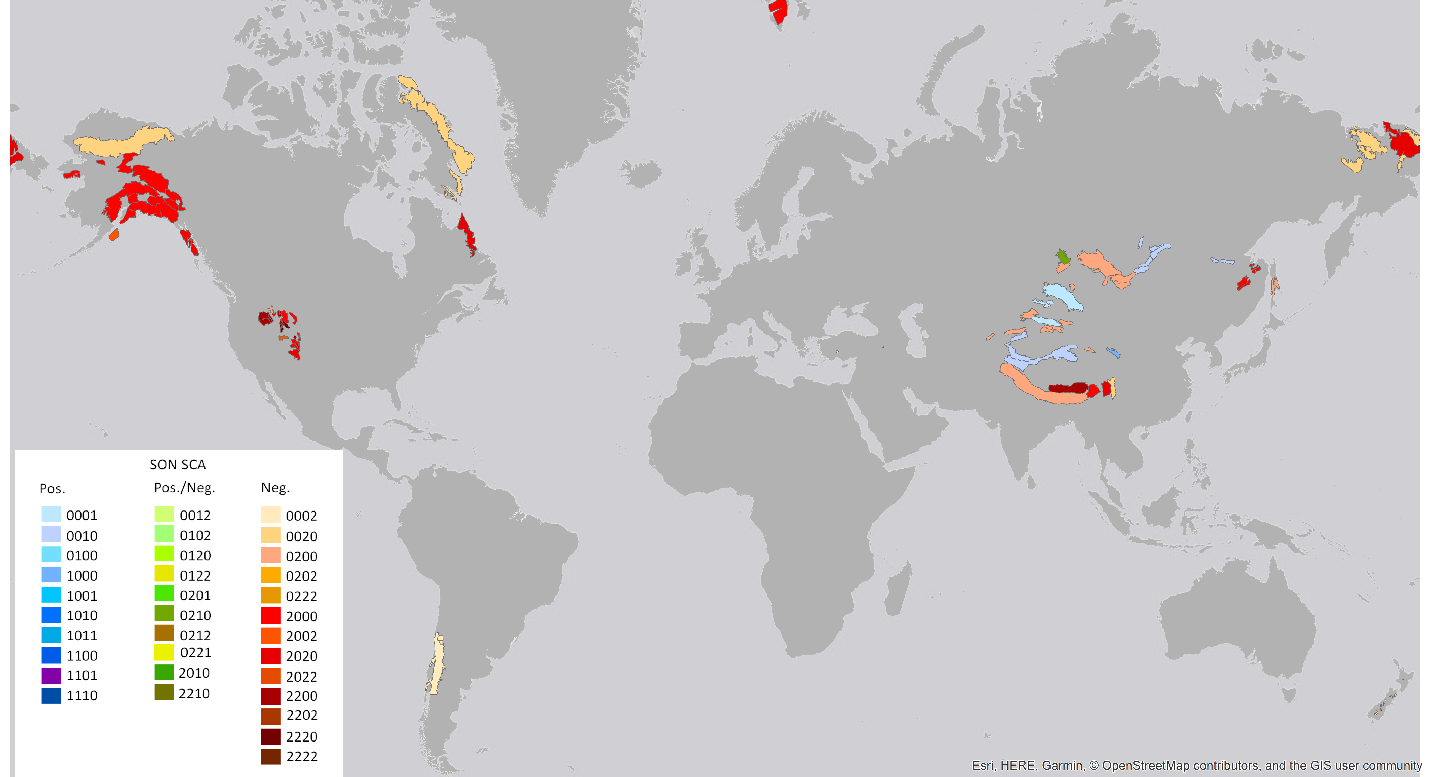


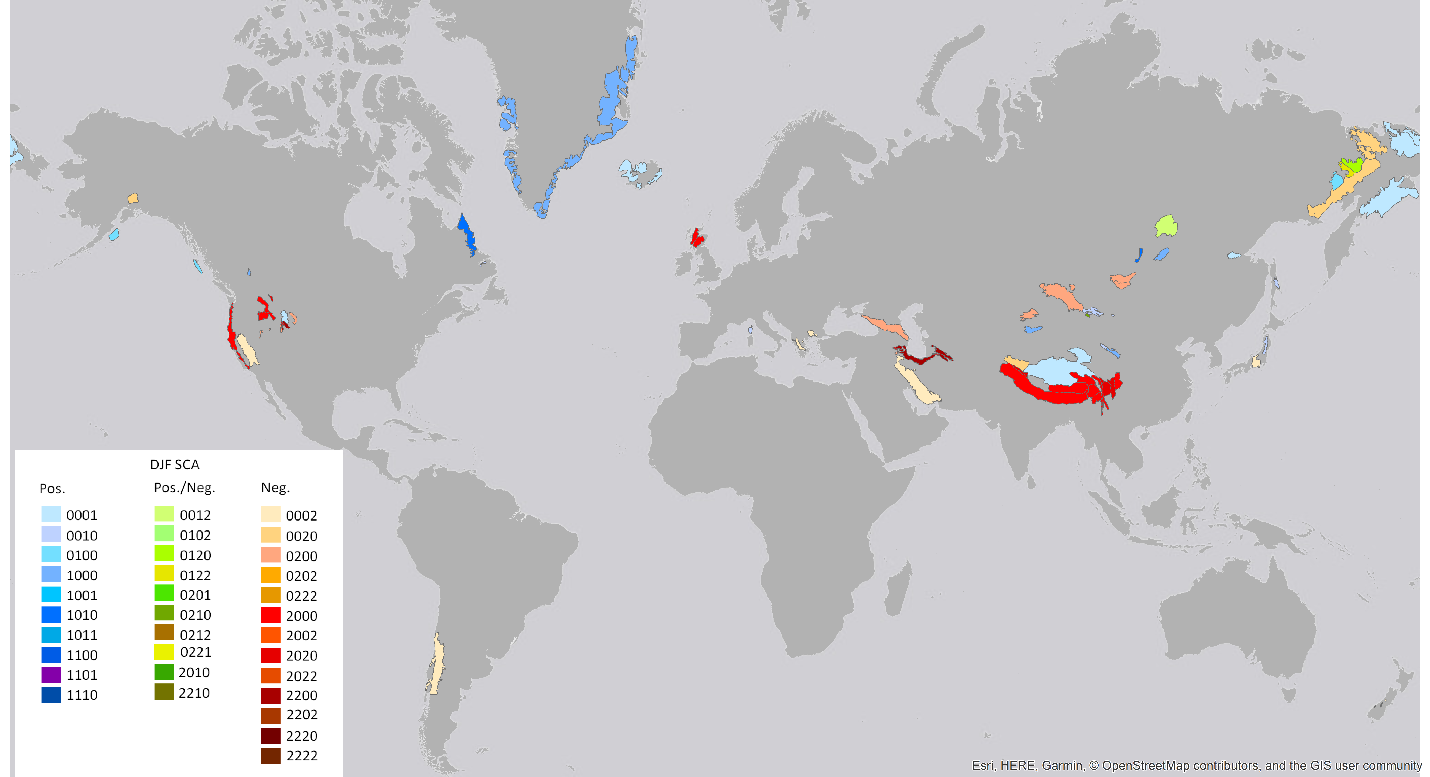


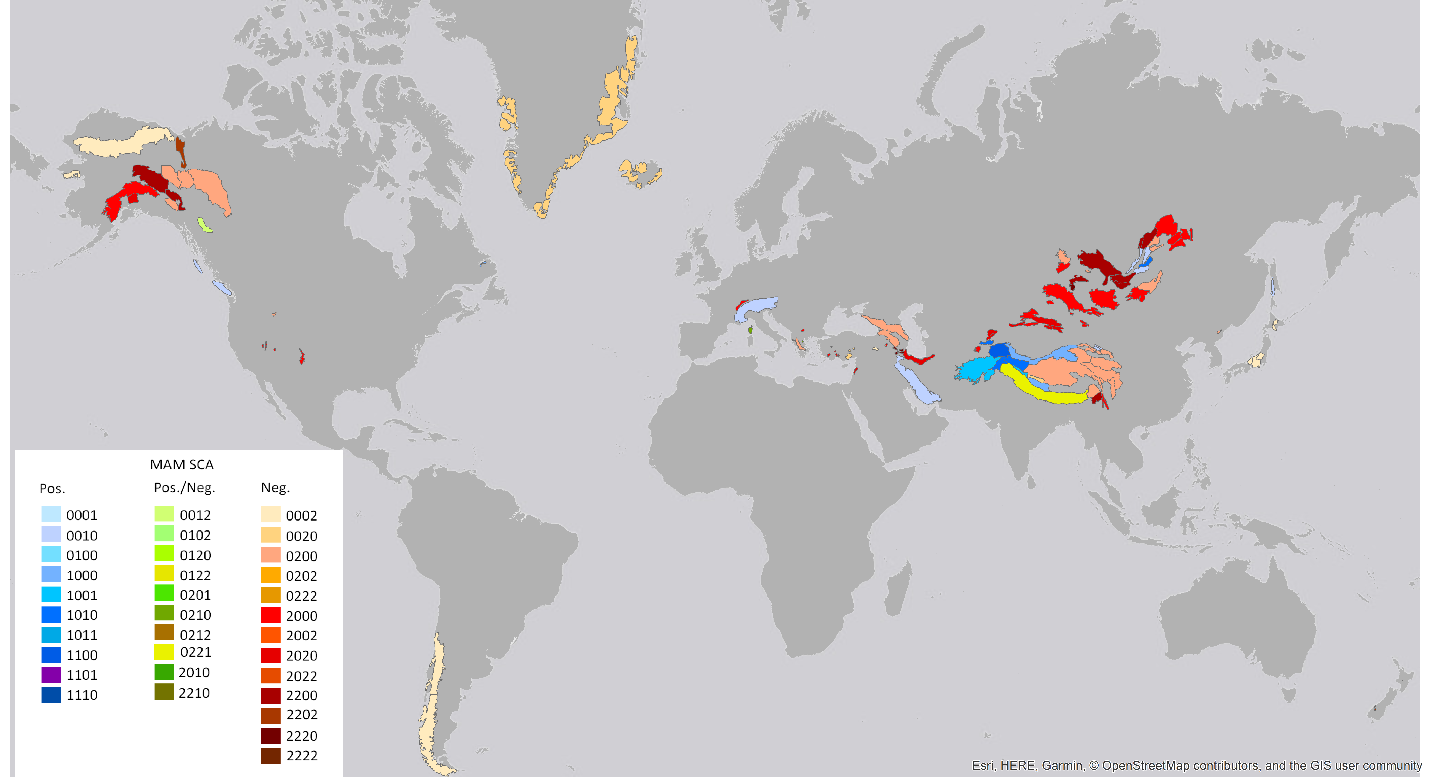


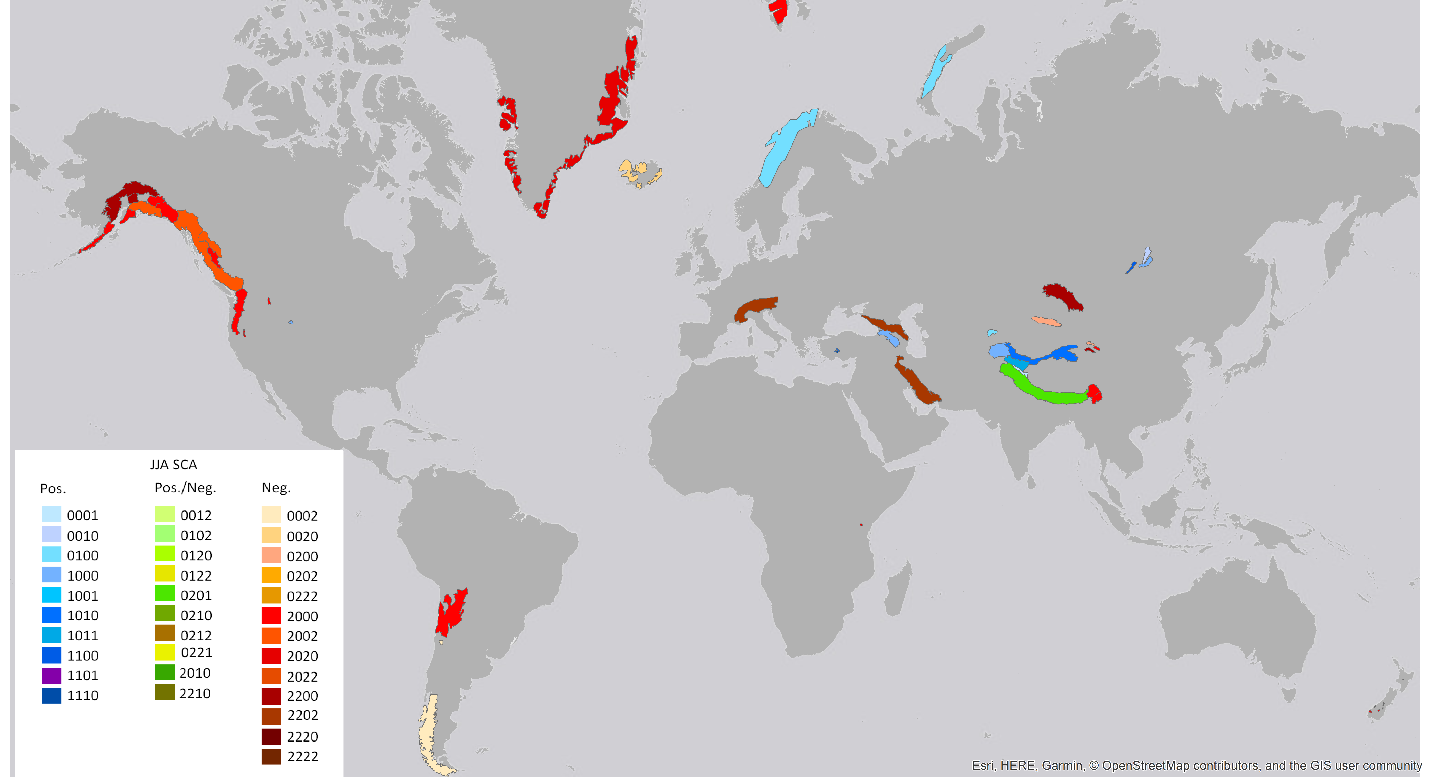


**Figure 2S:** Behaviour of the mountain areas for trends in SCA (SON, DJF, MAM, JJA) in different periods. The four digits in the legend represent the four analyzed time spans: the digit of thousands indicates significant trends in 1982-2020, the digit of hundreds in 1982-2000, the digit of tens in in 1990-2010, the single digit in 2000-2020. The value “2” indicates negative significant trends and the value “1” represents positive significant trends. For example: “0002” indicates a negative trend for the last period 2000-2020 and no significant trends in the other analyzed period. “2000” indicates a negative significant trend for the period 1982-2020 and no significant trends in the other analyzed periods (the maps were created by the author using the software ARCGIS v.10.1, www.esri.com).

**
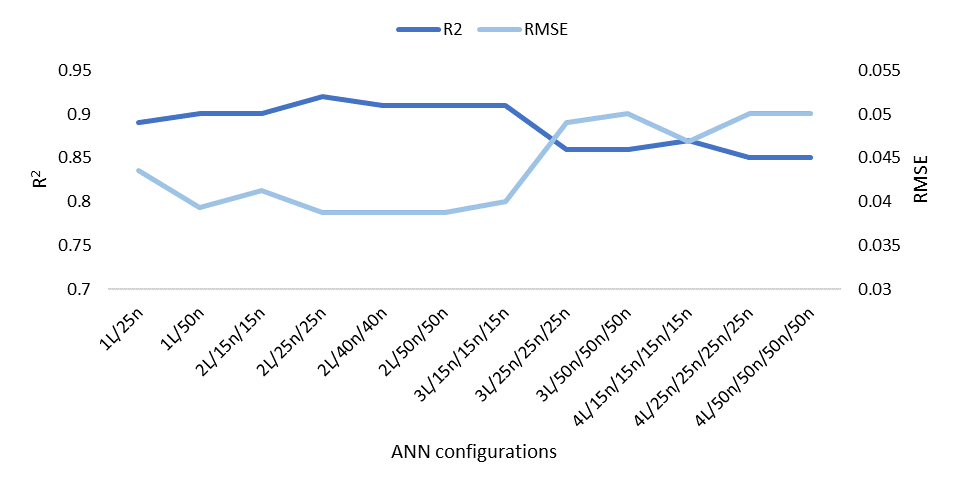
**

**Figure 3S:** Performances of different ANN configurations. In the x-axis the architecture configuration is represented as follows: 1L, 2L, 3L indicate that the structure has 1, 2 or 3 internal layers respectively. XXn indicates the number of neurons for each layer. For example, 2L/25n/25n indicates a structure with two internal layers and each layer with 25 neurons.
